# Differential Adhesion Molecule Expression during Murine Embryonic Stem Cell Commitment to the Hematopoietic and Endothelial Lineages

**DOI:** 10.1371/journal.pone.0023810

**Published:** 2011-09-06

**Authors:** Basha L. Stankovich, Esmeralda Aguayo, Fatima Barragan, Aniket Sharma, Maria G. Pallavicini

**Affiliations:** School of Natural Sciences, University of California Merced, Merced, California, United States of America; Instituto Nacional de Câncer, Brazil

## Abstract

Mouse embryonic stem cells (ESC) make cell fate decisions based on intrinsic and extrinsic factors. The decision of ESC to differentiate to multiple lineages *in vitro* occurs during the formation of embryoid bodies (EB) and is influenced by cell-environment interactions. However, molecular mechanisms underlying cell-environmental modulation of ESC fate decisions are incompletely understood. Since adhesion molecules (AM) influence proliferation and differentiation in developing and adult tissues, we hypothesized that specific AM interactions influence ESC commitment toward hematopoietic and endothelial lineages. Expression of AM in the adherens, tight and gap junction pathways in ESC subpopulations were quantified. E-cadherin (E-cad), Claudin-4 (Cldn4), Connexin-43 (Cx43), Zona Occludens-1 (ZO-1) and Zona Occludens-2 (ZO-2) transcript levels were differentially expressed during early stages of hematopoietic/endothelial commitment. Stable ESC lines were generated with reduced expression of E-cad, Cldn4, Cx43, ZO-1 and ZO-2 using shRNA technology. Functional and phenotypic consequences of modulating AM expression were assessed using hematopoietic colony forming assays, endothelial sprouting assays and surface protein expression. A decrease in E-cad, Cldn4, Cx43 and ZO-1 expression was associated with less commitment to the hematopoietic lineage and increased endothelial differentiation as evidenced by functional and phenotypic analysis. A reduction in ZO-2 expression did not influence endothelial differentiation, but decreased hematopoietic commitment two-fold. These data indicate that a subset of AM influence ESC decisions to commit to endothelial and hematopoietic lineages. Furthermore, differentially expressed AM may provide novel markers to delineate early stages of ESC commitment to hematopoietic/endothelial lineages.

## Introduction

Stem cells from multiple sources are used for transplantation therapy and tissue regeneration. For example, endothelial progenitor cells (EPC) are used to treat tissue ischemia, repair blood vessels and relieve pulmonary hypertension in diabetes, vascular and kidney diseases [1]. Hematopoietic stem cells (HSC) have been used to treat blood disorders and influence immunological tolerance in graft versus host disease [2]. Unfortunately, it is difficult to obtain sufficient quantities of EPC or HSC for therapy by *in vitro* expansion of these populations [1], [3]. Embryonic stem cells (ESC) are capable of indefinite self-renewal and under appropriate culture conditions may potentially offer an infinite supply of progenitors. However, the ability to reliably guide ESC toward hematopoietic or endothelial lineages is complicated by a lack of understanding of key regulatory signals/pathways involved in their proliferation and differentiation decisions. Increased understanding of factors that guide early stages of ESC commitment decisions towards hematopoietic and endothelial lineages is an important step in developing strategies to direct differentiation.

Embryoid bodies (EB), generated from ESC after removal of leukemia inhibitory factor (LIF), are comprised of cells contributing to multiple lineages [4]. EB that promote hematopoietic and endothelial differentiation of ESC *in vitro* are propagated in liquid culture or methylcellulose [5], [6]. However, the frequency of endothelial and hematopoietic cells in these EB is extremely low (9% CD34-expressing cells in day 8 murine EB [7]). Preferential induction of ESC commitment to multiple different lineages can be accomplished by varying culture conditions (refer to Keller [5] for review). However, in the absence of exogenously added cytokines that support hematopoietic commitment, EB generate low numbers of hematopoietic and endothelial cells. Even in the presence of a cytokine/growth factor-rich medium designed to promote differentiation [8], ESC produce low numbers of hematopoietic and endothelial cells. Propagation of stem cells from fetal or adult hematopoietic tissues *in vitro* using differentiating inducing cytokines invariably results in exhaustion of the expansion capabilities of the stem cell population. Developing an understanding of cell-cell and cell-environment interactions that guide ESC towards hematopoiesis and endothelial cell commitment may provide opportunities for increased expansion of hematopoietic stem cells derived from ESC.

Junction proteins comprise one family of adhesion molecules (AM) expressed in ESC. Several connexins, including Connexin-43 (Cx43), form functional gap junctions when ESC are maintained in an undifferentiated state; Cx43 is down-regulated during differentiation [9], [10]. Disruption of E-cadherin (E-cad), an adherens junction protein, perturbs the formation of EB [11]. Junction associated proteins, such as Zona Occludens-1 and -2 (ZO-1 and ZO-2) are expressed in both mouse ESC [12] and endothelial cells. While junction proteins are expressed during EB development, their role in hematopoietic and endothelial commitment decisions of ESC is not well established. We explored the role of adhesion molecules, and/or their downstream signaling or effector molecules in specification of ESC to hematopoietic and endothelial lineages.

In this study, we quantified AM expression during EB formation and lineage commitment in the absence of exogenous lineage-specific cytokines and assessed phenotypic and functional consequences of modulating levels of selected AM. We compared transcript expression profiles of a panel of AM genes, shown previously to be expressed in differentiating EB [10], [12], [13]. Quantitative analysis of AM gene expression profiles was performed on Bry, Flk-1 and Scl expressing subpopulations, representing early hematopoietic/endothelial commitment stages of EB. E-Cad, Cx43, ZO-1 and ZO-2 were among the genes that were differentially expressed during lineage commitment. The extent to which the AM encoded by these genes modulate ESC commitment to hematopoietic and endothelial lineages was assessed using RNAi technology coupled with functional assays. These data are the first to demonstrate that modulation of AM expression in ESC perturbs early hematopoietic and endothelial commitment decisions. Furthermore, we show that differentially expressed AM may be useful to further discriminate phenotypes of transitional stages of early hematopoietic and endothelial differentiation.

## Results

### AM are differentially expressed during EB development

**Figure 1:**
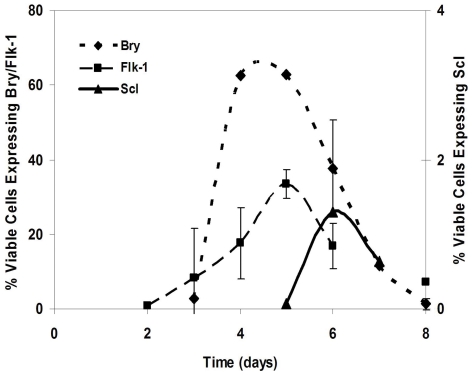
Expression of markers of early transitional stages of hematopoietic and endothelial differentiation is temporally regulated. Bry-GFP, D3-ESC and Scl-LacZ ESC were differentiated in EB media for 8 days and analyzed flow cytometrically for expression of Bry, Flk-1 and Scl, respectively. Non-viable cells were excluded based on propidium iodide (PI) fluorescence. Error bars represent standard deviation with n = 3.

**Figure 2:**
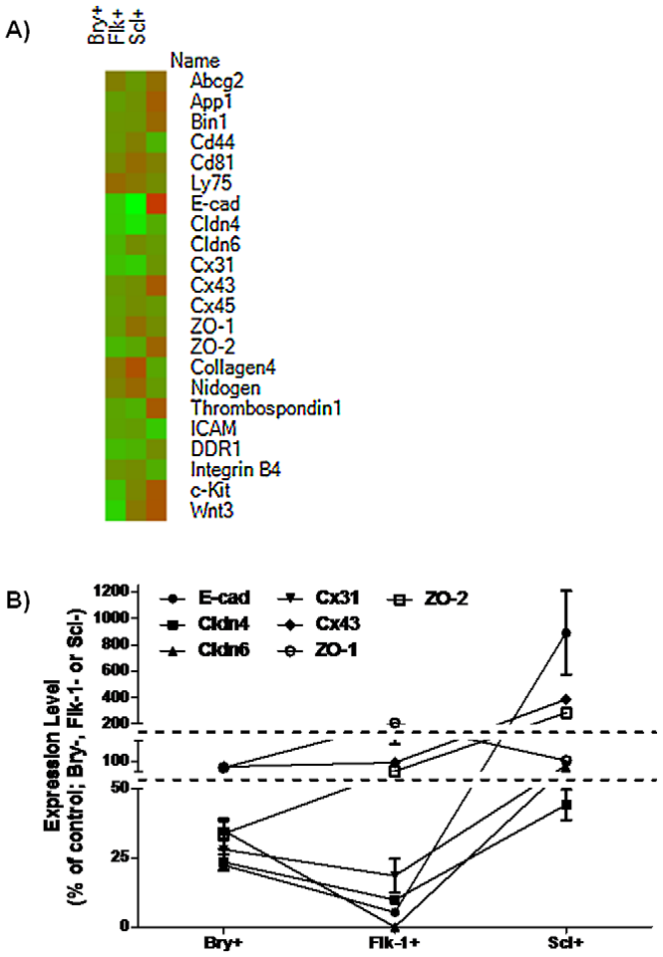
AM are differentially expressed in early hematopoietic and endothelial subpopulations of developing EB. Quantitative analysis of gene expression was performed on populations of ESC differentiated in EB. Cells were sorted by FACS based on expression of Bry for mesodermal commitment, Flk-1 for hematopoietic and endothelial precursors and Scl for early hematopoietic cells. RNA was extracted and quantitative RT-PCR was performed. A) 21 genes were analyzed. Heat map was generated with Heatmap Builder (http://ashleylab.stanford.edu/tools_scripts.html). B) Samples are normalized to GAPDH and relative quantification is reported as percent of control (expression from sorted cell populations negative for expression of Bry, Flk-1 and Scl, respectively). Dashed line represents 2-fold increase/decrease in transcript expression level. (n = 3 for each sample; error bars represent standard deviation).

Gene expression profiles of a panel of AM were measured in developing EB (Table S1). Since developing EB are comprised of multiple lineages, we used differentiation associated markers and multivariate flow cytometric analysis to discriminate and sort Bry+, Flk-1+ and Scl+ subpopulations prior to quantifying AM gene expression using qPCR. These populations were discriminated using Bry-linked GFP expression, Scl-linked LacZ expression and a Flk-1-specific monoclonal antibody. Bry, Flk-1 and Scl expression levels in EB peaked at day 4, 5 and 6, respectively (Figure 1). Subpopulations were isolated at time periods when Bry, Flk-1 and Scl were highly expressed. Thus, committed mesoderm GFP+ cells in Bry-GFP EB were sorted at day 4, Flk-1+ cells undergoing early hematopoietic and endothelial differentiation were sorted from day 5 EB generated from D3-ESC, and Scl-expressing subpopulations, containing cells that are committed to hematopoiesis, but have not fully differentiated, were sorted from day 6 EB generated from Scl-LacZ ESC. Transcript levels of 21 cell adhesion-associated genes in the sorted populations were differentially expressed in one or more subpopulations (Table 1; Figure 2A). Expressed genes in the Bry+, Flk-1+ or Scl+ subpopulations were categorized according to Gene Ontology (GO) classification. Junction molecules were differentially expressed with the greatest frequency (62% of populations screened) in comparison to remaining GO categories, in which 13–58% of populations expressed 2-fold or greater increased/decreased transcript levels. Junction molecule expression in Bry+, Flk-1+ and Scl+ subpopulations varied 9–20 fold compared to cells that did not express Bry, Flk-1 or Scl. For example, E-cad expression was 20-fold less in Flk-1+ cells in day 5 EB compared to cells that did not express Flk-1 in the same EB. E-cad was 9-fold higher in Scl+ cells compared with Scl− cells in day 6 EB (Figure 2B). These data suggest that E-cad, Cx43, ZO-1 and ZO-2 expression are associated with commitment. While these data suggest a relationship between expression of these AM and commitment, the functional role of these AM in the commitment decisions were evaluated using functional assays.

**Table 1:** Adhesion molecules are differentially expressed during hematopoiesis.

	Gene	Bry+	Flk-1+	Scl+
**Membrane** **Associated**	**Abcg2**	N/S	N/S	2.27+/−0.51
	**App1**	N/S	N/S	3.43+/−0.13
	**Bin1**	N/S	N/S	2.57+/−1.81
**Hematopoietic** **Lineage**	**Cd44**	N/S	N/S	0.37+/−0.01
	**Cd81**	N/S	2.32+/−0.13	N/S
	**Ly75**	2.46+/−0.04	N/S	N/D
**Junction** **Molecule**	**E-cad**	0.22+/−0	0.05+/−0.01	8.93+/−3.18
	**Cldn4**	0.23+/−0.03	0.1+/−0	0.44+/−0.05
	**Cldn6**	0.35+/−0.03	N/D	N/S
	**Cx31**	0.28+/−0.08	0.19+/−0.06	N/S
	**Cx43**	N/S	N/S	3.86+/−0.28
	**ZO-1**	N/S	2.07+/−0.22	N/S
	**ZO-2**	0.34+/−0.06	0.52+/−0.02	2.83+/−0.1
**Extracellular** **Matrix**	**Collagen4**	N/S	4.64+/−0.16	0.51+/−0.03
	**Nidogen**	N/S	2.56+/−0.28	N/S
	**Thrombospondin1**	0.54+/−0.07	0.39+/−0.04	3.74+/−0.46
**Cell Adhesion** **Molecule**	**ICAM**	0.56+/−0.09	N/S	0.21+/−0.07
**Receptor-** **Ligand**	**Ddr1**	0.3+/−0.01	0.37+/−0.02	N/D
	**Integrin B4**	N/S	N/D	0.41+/−0.03
	**c-Kit**	0.28+/−0.01	N/S	4+/−0.16
	**Wnt3**	0.16+/−0.01	N/S	4.15+/−1.46

Transcript expression profiles of AM were generated using RT-qPCR from discrete transitional stages of early hematopoietic/endothelial development. Expression levels in one or more subpopulations, Bry+, Flk-1+ and Scl+, were up- or down-regulated 2-fold or greater relative to Bry−, Flk-1− and Scl− subpopulations, respectively. (N/D = not determined; N/S = not significant; greater than 2-fold difference).

### ESC lines with down-regulated expression of junction molecules

The functional role of E-cad, Cx43, ZO-1 and ZO-2 in ESC commitment decisions was investigated by modulating gene expression levels using RNAi technology and assessing the consequences of the modulation using functional assays. D3-ESC were modified using lentiviral constructs with shRNA sequences specific to E-cad, Cx43, ZO-1 and ZO-2. Each construct expressed GFP to fluorescently label cells infected with lentiviral particles (Figure S1). Knockdown cells lines, Kd-E-cad, Kd-Cx43, Kd-ZO1 and Kd-ZO2, were characterized at transcript and protein levels and evaluated for retention of pluripotency. Transduced GFP+ cells comprised greater than 95% of the viable cell population for multiple generations (Figure S2A) for each construct. GFP expression was also stable for multiple generations. To confirm that modulation of these AM did not affect pluripotency of ESC, expression of SSEA-1 and Oct-4, markers associated with murine ESC pluripotency, were quantified flow cytometrically. SSEA-1 and Oct-4 were expressed at similar levels in D3-ESC and engineered ESC lines propagated in ESC medium (Figure S2C, S2D).

**Figure 3:**
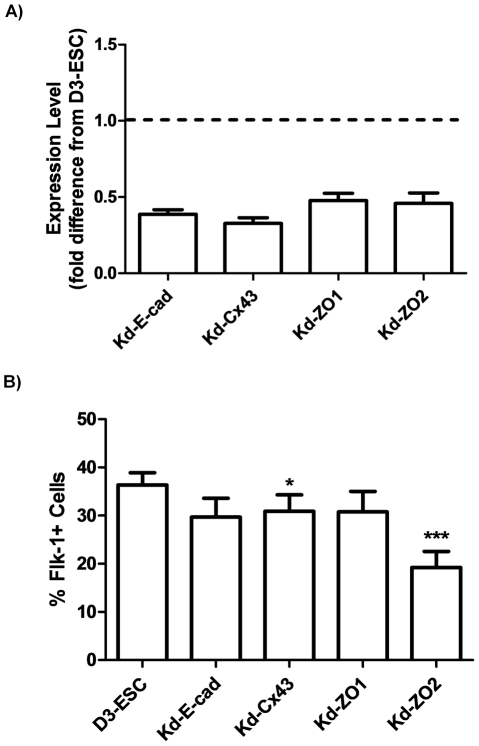
ESC with knockdown of junction molecules by shRNA maintain ability to differentiate to Flk-1 cells. A) shRNA constructs efficiently silence endogenous mRNA in mESC. Quantitative RT-PCR was performed on GFP expressing cells generated with lentiviral constructs containing shRNA specific to E-Cadherin, Cx43, ZO1 and ZO2 to analyze transcript expression level. Samples were normalized to GAPDH and expressed relative to transcript levels in untransfected D3-ESC (dashed line). Error bars represent standard deviation of n = 3 samples. B) Flk-1 expression does not significantly differ in knockdown ESC lines. ESC lines stably expressing shRNA sequences specific to E-Cad, Cx43, ZO-1 and ZO-2 and ESC lines with increased knockdown levels of E-Cad and ZO-1 were differentiated in EB for 5 days and Flk-1 expression was analyzed flow cytometrically using anti-Flk-1 conjugated to PE. Non-viable cells were excluded based on fluorescence of DAPI. Paired t-tests with D3-ESC were performed with n = 7 (Kd-E-cad, Kd-Cx43, Kd-ZO-1) or n = 6 (Kd-ZO-2). * p<0.05; *** p<0.0005. Error bars represent standard error of the mean.

AM transcript expression levels were quantified in engineered ESC lines using RT-qPCR (Figure 3A). The expression of E-cad, Cx43, ZO-1 and ZO-2 was reduced in transduced ESC populations compared to untransduced D3-ESC approximately 2–4-fold. Western blot analysis confirmed decreased expression of E-cad, Cx43 and ZO-2 at the protein level in knockdown ESC lines (Figure S3A). Inadequate antibody quality precluded confirmation of the knockdown of ZO-1 at the protein level; even through transcript was clearly reduced. Specificity of shRNA effect was verified by qPCR of target gene expression in ESC expressing scrambled shRNA sequences (Scram-GFP) (Figure S3B). Expression of scrambled shRNA in ESC did not reduce expression of E-cad, Cx43, ZO-1 or ZO-2.

### Knockdown of junction molecules influence differentiation decisions in ESC

#### Hematopoietic/Endothelial Progenitors

We next examined effects of modulating E-cad, Cx43, ZO-1 and ZO-2 levels in ESC differentiation toward hematopoietic and endothelial lineages. Perturbations in early hematopoietic/endothelial commitment were assessed by quantifying numbers of cells committing to a hematopoietic/endothelial progenitor, Flk-1+ progenitor. The frequency of Flk-1+ progenitors in engineered ESC with reduced E-cad, Cx43 and ZO-1 levels in day 5 EB was similar to controls (Figure 3B). The temporal profile of Flk-1 expression over a 6 day period in the engineered ESC lines was also comparable to D3-ESC (data not shown). In contrast, reduced ZO-2 expression in Kd-ZO2 ESC resulted in approximately a 2-fold decrease in the Flk-1+ progenitor population in day 5 EB compared to D3-ESC. These data suggest that while E-cad, Cx43 and ZO-1 levels have minimal effects on the frequency of Flk-1+ progenitor cells, ZO-2 may be involved in the commitment to Flk-1+ progenitor cells.

#### Hematopoietic differentiation

**Figure 4:**
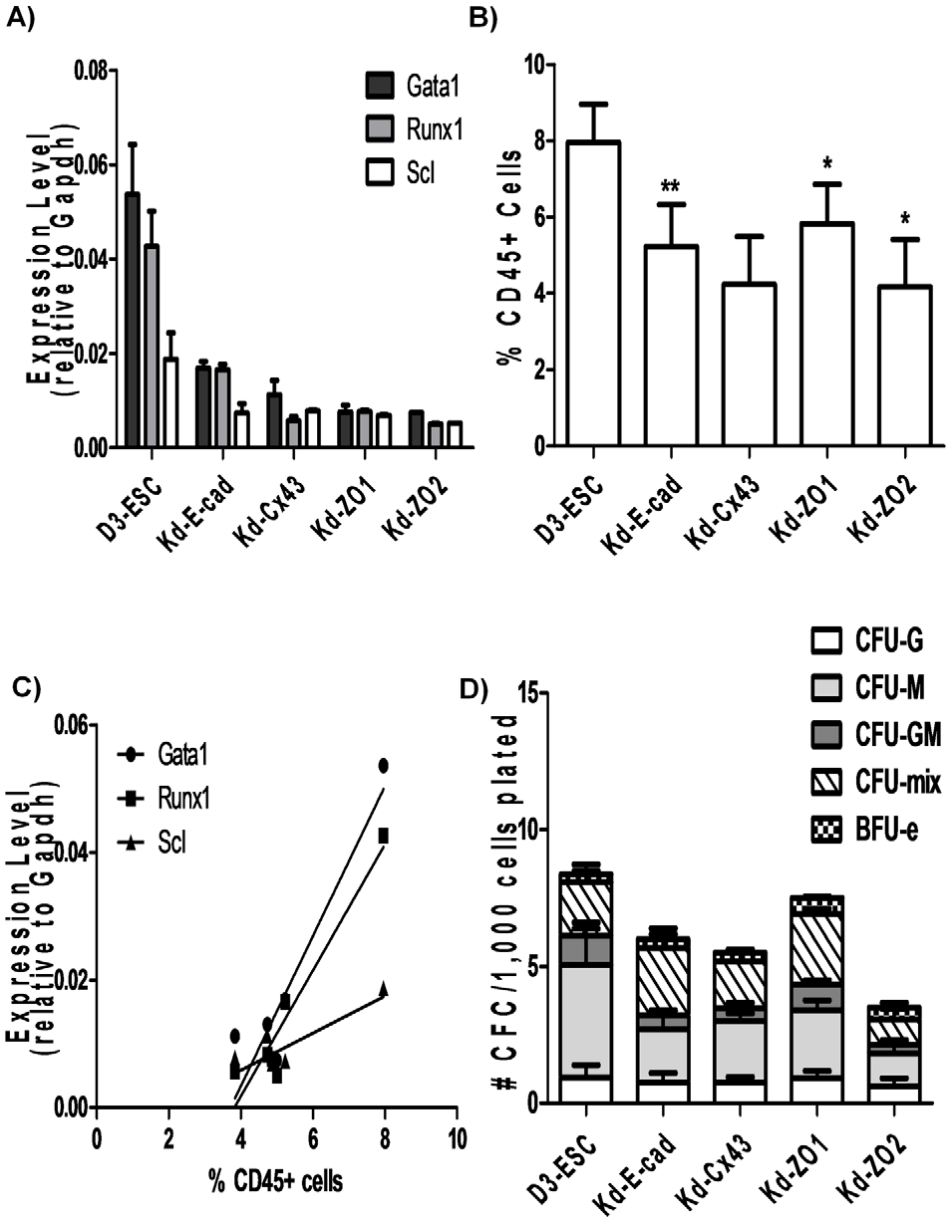
Hematopoietic potential decreases in ESC with down-regulation of junction molecules. A) Knockdown ESC lines were differentiated in EB for 9 days. RNA was extracted from GFP+ cells and quantitative RT-PCR was performed using SYBR Green and primers for hematopoietic transcription factors Gata1, Runx1 and Scl. Error bars represent standard deviation of n = 3 samples. B) ESC lines stably expressing shRNA sequences specific to E-cadherin, Cx43, ZO-1 and ZO-2 were differentiated in EB for 11 days. CD45 expression was analyzed flow cytometrically using anti-CD45 conjugated to PerCP-Cy5.5. Non-viable cells were excluded based on DAPI fluorescence. Paired t-tests were performed with D3-ESC for n = 7 (Kd-Cx43) and n = 6 (Kd-E-cad, Kd-ZO1 and Kd-ZO2) (* p<0.05; ** p<0.005). Error bars represent standard error of mean. C) TF expression level linearly correlates with decreased frequency of CD45+ cells. Gata1: R^2^ = 0.87; Runx1: R^2^ = 0.91; Scl: R^2^ = 0.69. D) Day 5 EB with knockdown of E-cadherin, Cx43, ZO-1 and ZO-2 were dissociated and replated as single cells to determine colony forming potential. Hematopoietic colonies were scored after 12 days in methylcellulose media for presence of granulocyte colony forming units (CFU-G), macrophage colony forming units (CFU-M), erythroid burst forming units (BFU-E), bipotent progenitors of granulocyte and macrophages (CFU-GM) and multipotent progenitors of these myeloid lineages (CFU-mix). Error bars represent standard error of the mean for n = 4 plates.

Evidence that modulation of AM expression influences hematopoietic commitment was investigated using 3 endpoints: expression of hematopoietic TF, Runx1, Gata1 and Scl, identification and enumeration of CD45+ hematopoietic cells, and myeloid colony forming assays. The expression of hematopoietic TF was quantified in developing EB (Figure 4A). A decrease in E-cad expression resulted in a 3-fold reduction in Runx1, Gata1 and Scl expression. Reduced levels of Cx43 resulted in 2–8-fold decreases in levels of Runx1, Gata1 and Scl. Knockdown of ZO-1 and ZO-2 reduced hematopoietic TF expression 3–7-fold and 4–9-fold, respectively. The frequency of cells expressing CD45, an extracellular marker expressed on the surface of most hematopoietic cells, is an indication of hematopoietic differentiation commitment. Reduced expression of E-cad, Cx43, ZO-1 and ZO-2 levels was associated with an approximately 2-fold reduction in the frequency of CD45+ cells (Figure 4B). Additionally, the frequency of CD45+ cells in day 11 EB is linearly correlated with expression levels of hematopoietic TF (Figure 4C). Another indicator of hematopoietic commitment is the ability to form myeloid colonies. The number of colony forming units (CFU) was decreased approximately 2-fold in ESC lines with reduced E-cad, Cx43 and ZO-2 compared to D3-ESC (Figure 4C). The decrease was most pronounced in cells undergoing macrophage commitment (CFU-M). The frequency of CFU-M was decreased in Kd-E-cad and Kd-Cx43 approximately 2-fold and in Kd-ZO2 greater than 3-fold. These data suggest hematopoietic commitment is influenced by expression of these junction molecules.

#### Endothelial differentiation

**Figure 5:**
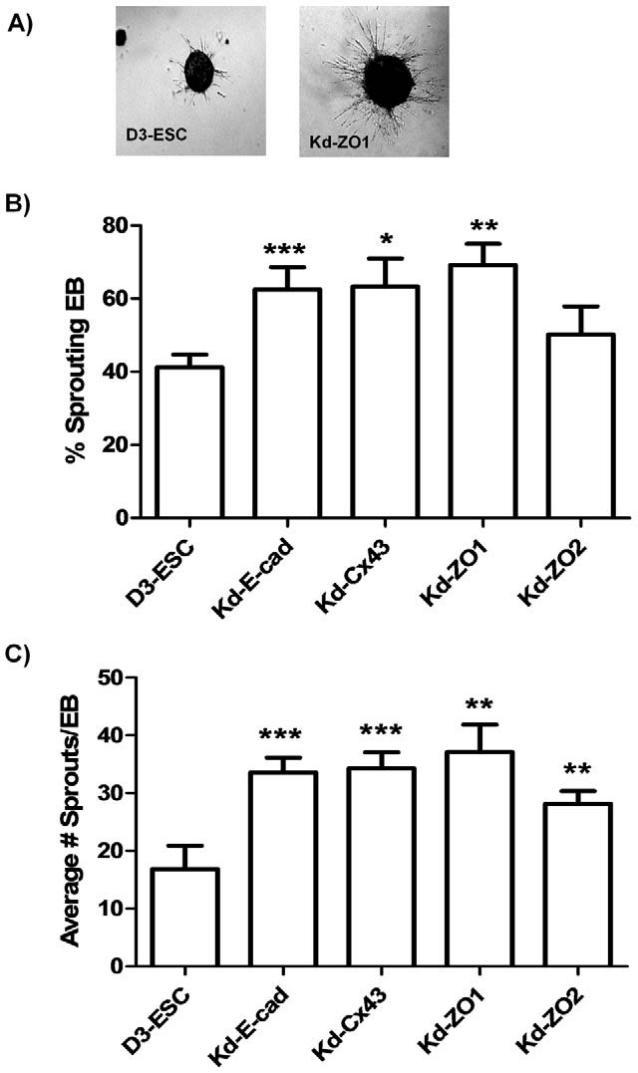
Endothelial commitment is increased in ESC knockdown lines. A) ESC lines stably expressing shRNA sequences specific to E-cadherin, Cx-43, ZO-1 and ZO-2 were differentiated in Collagen I with 100 ng/ml bFGF and 50 ng/ml VEGF for 11 days. B) Percentage of sprouting EB and C) number of sprouts per sprouting EB were manually measured on photomicrographs in ESC reduced in expression of junction molecules compared to D3-ESC control. *** p-value<0.0005, ** p-value<0.005; * p-value<0.05.

Quantitative measurements of two endothelial associated receptors (Tie1 and Tie2), as well as endothelial sprouting assays, were used to assess effects of AM manipulation on endothelial development. Tie1 expression was decreased approximately 2-fold in Kd-ZO2; whereas expression in the other Kd-ESC lines was similar to control D3 (data not shown). Tie2 expression was similar to D3-ESC for Kd-ESC lines. Differentiation of ESC in the presence of endothelial lineage-specific cytokines generates EB with sprouts (Figure 5A). Reduction of E-cad, Cx43 and ZO-1 expression increased the frequency of EB with endothelial sprouting approximately 2-fold (Figure 5B), and the number of sprouts on each EB by approximately 2-fold (Figure 5C). Knockdown of ZO-2 did not significantly influence the frequency of EB generating endothelial sprouts; however, there was an approximately 2-fold increase in number of sprouts on each EB. Tie1/2 data and the sprouting assays suggest that E-cad, Cx43, ZO-1 and ZO-2 expression influence endothelial cell differentiation from ESC derived progenitors.

## Discussion

Cell-cell interactions are known to regulate transcriptional machinery for proliferation and development in multiple systems [14], [15], [16]. However, the contributions of cell surface mediated protein signaling in ESC fate decisions are incompletely understood. Understanding the molecular signaling that influences commitment decisions is important in developing strategies to guide ESC commitment for regenerative therapies. Since AM are known to have major roles in cell differentiation during embryonic development [17], [18], [19], [20], we explored the contributions of AM in ESC commitment decisions to hematopoietic/endothelial lineages. We describe expression of 21 genes encoding extracellular matrix proteins, cell adhesion molecules (CAM), hematopoietic lineage markers, junction molecules, receptors and other membrane associated proteins in ESC proliferation/differentiation and the functional consequences of manipulating AM expression levels on hematopoietic and endothelial commitment. Previous studies of expression profiling, using microarray and proteomic analysis, of murine ESC identified expressed AM [10], [12], [13] in ESC. While these data demonstrate the AM are expressed during ESC culture, the functional role of individual AM in influencing ESC commitment decisions was not investigated. The ability to effectively direct differentiation of ESC for therapy will require understanding the complex cell-environment signaling pathways that influence commitment decisions. ESC differentiation towards the hematopoietic lineage is challenging, typically generating only low numbers of hematopoietic cells [7]. While hematopoietic commitment can be increased by addition of cytokines [8], hematopoietic differentiation is still low and, addition of cytokines often drives differentiation beyond stem/progenitor stages, thus reducing usefulness for sustained therapy. Since AM are differentially expressed after LIF removal, we explored whether manipulation of AM expression influenced ESC commitment to hematopoietic and endothelial lineages.

EB are comprised of multiple transitional stages and lineages [4], thus reduction of heterogeneity by subpopulation isolation facilitates exploration of AM contributions to early hematopoietic/endothelial lineage commitment. Early hematopoietic/endothelial differentiation proceeds through defined stages of commitment. ESC undergoing commitment to the mesoderm lineage express the TF Bry [21], [22]. A VEGF receptor, Flk-1, is expressed in a subpopulation of Bry+ cells which are progenitors to early endothelial and hematopoietic cells [23], [24] and Flk-1+ cells that express Scl are committed to hematopoiesis [25]. We used Bry, Flk-1 and Scl expression to discriminate subpopulations in developing EB. We show that temporal oscillations in Bry, Flk-1 and Scl expression during EB development are consistent with those reported previously [22], [26], [27].

Junction molecules, including E-cad, Cx43, ZO-1 and ZO-2, were among the 21 AM whose levels changed preferentially in EB subpopulations expressing Bry, Flk-1 or Scl. E-cad is present in adherens junctions, which indirectly link the cytoskeletons of adjacent cells [28]; ZO-1 and ZO-2 are associated with tight junction complexes, which generate a permeability barrier and activate intracellular signaling responses that suppress cellular proliferation [15]; Cx43 forms gap junctions, which allow second messengers to pass between cells through the cytosol [9]. The temporal and quantitative variation of E-cad, Cx43, ZO-1 and ZO-2 expression during EB formation in culture conditions that support hematopoietic and endothelial commitment suggest a role for these proteins in ESC differentiation.

The effects of modulating E-cad, Cx43, ZO-1 and ZO-2 expression on hematopoietic and endothelial commitment potential of manipulated ESC were investigated to establish genotype-phenotype relationships of these AM. Knockdown ESC lines were engineered using RNAi stably introduced into the ESC genome using lentiviral transduction. Although clonal analysis is preferable in determining the role of AM in hematopoietic/endothelial commitment, culture of ESC at low density often results in loss of pluripotency. To reduce the heterogeneity in lentiviral transduction efficiency, transduced ESC were sorted based on GFP expression to generate ESC lines with greater than 95% of cells containing incorporated shRNA sequences. Using engineered ESC lines, we demonstrated that reduced levels of E-cad, Cx43, ZO-1 and ZO-2 had minimal influence on the frequency of SSEA-1 expressing cells or level of Oct-4 expression, markers associated with ESC pluripotency. These data suggest that pluripotency (as assessed by SSEA-1 and Oct-4 expression) is unaffected by manipulation of these AM.

**Figure 6:**
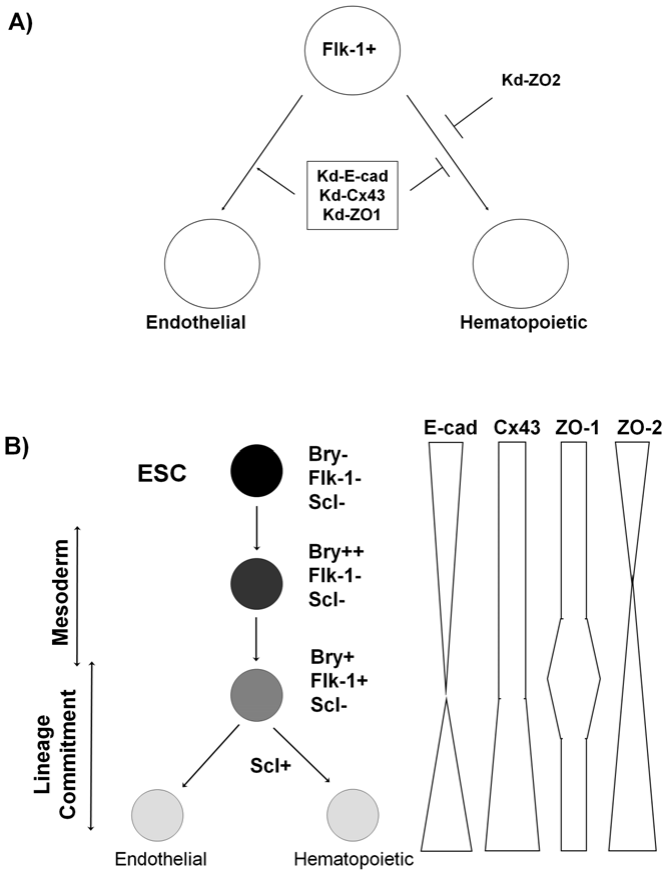
Adhesion molecules expression and influence in hematopoietic and endothelial differentiation. A) Knockdown of junction molecules ZO-1, Cx43 and E-Cad in murine ESC results in increased commitment to differentiate along the endothelial lineage with concurrent loss of commitment to hematopoietic cells. These fate decisions occur after the emergence of Flk-1 precursors in EB. Knockdown of ZO-2 in mouse ESC reduces hematopoietic commitment without influencing endothelial differentiation. B) Hematopoietic stages of differentiation can be identified using intracellular and extracellular markers. ESC undergoing mesodermal commitment express the transcription factor Bry. As mesodermal cells become committed to early hematopoietic and endothelial progenitors, the cell surface receptor, Flk-1, is expressed. Scl is a transcription factor expressed as cells commit specifically to the hematopoietic lineage. Expression of E-cad, Cx43, ZO-1 and ZO-2 varies during early stages of hematopoietic and endothelial differentiation of ESC.

If AM modulations affect the decision of ESC to differentiate into a Flk-1+ progenitor, then the number and/or timing of Flk-1 expressing engineered ESC during EB development would be altered. A reduction in E-cad, Cx43 and ZO-1 expression did not appear to affect the frequency of Flk-1+ cells. However, these same AM appeared to have a more prominent role in influencing intermediate and later stages of hematopoietic commitment because Kd-E-cad, Kd-Cx43 and Kd-ZO1 ESC showed reduced expression of hematopoietic TF, as well as a lower frequency of cells expressing CD45, present on cells committed to hematopoiesis [4] (Figure 4). These data suggest that Gata1, Runx1 and Scl expression, associated with intermediate stages of hematopoietic commitment are modulated by E-cad, Cx43 and ZO-1 expression. The reduction in TF expression in Kd-E-cad, Kd-Cx43 and Kd-ZO1 was paralleled with a decrease in the number of CFU, particularly CFU-M. Hematopoietic commitment decreased in engineered Kd-Cx43 ESC by approximately 2-fold, as evidenced by frequency of CD45+ cells and CFU. The reduction in hematopoietic commitment in Kd-Cx43 is consistent with data reported by Cancelas, et al. [29] who observed a reduction in hematopoiesis in fetal liver of Cx43^−/−^ embryos particularly CFU-GM and BFU-e (40–50% decrease). Hematopoiesis is not completely inhibited in Cx43^−/−^ embryos, suggesting interactions between multiple proteins and/or pathways in hematopoietic commitment. Since the knockdown of AM with shRNA sequences does not completely abrogate gene expression in ESC, a more extensive knockdown AM in ESC would be predicted to have a larger impact on hematopoietic/endothelial commitment. Although our knockdown was not complete (33% of control expression), we observed decreased frequency of CD45+ cells (48% of control), CFU-GM (48% of control) and BFU-e (68% of control), which is comparable to data reported by Cancelas et al. [29] Fok, et al reported that levels of E-cadherin, in heterozygous and homozygous knockout ESC, correlated with size of EB [11]. Similarly, ZO-1 knockdown in mouse morulas reduced blastocyst formation in a concentration dependent manner [30]. Overall, our ESC data suggest that E-cad, Cx43, and ZO-1 do not play substantial roles in hematopoietic commitment prior to the Flk-1+ progenitor stage (Figure 6B), but may be involved in differentiation decisions at or after the Flk-1+ expression stage.

Flk-1+ cells are also capable of differentiating to endothelial cells [22], [25], [31]. ESC propagated in a collagen matrix containing endothelial-specific cytokines differentiate along the endothelial lineage, forming EB with endothelial sprouts that invade the matrix and express the endothelial marker CD31 (PECAM) [32]. Invasion is used as a surrogate endpoint for angiogenesis [32]. Activators of angiogenesis, VEGF and FGF-2, increase the number of EB with endothelial sprouts, while inhibitors of angiogenesis decrease the length of endothelial sprouts on EB [32]. We show that reduced E-cad, Cx43 and ZO-1 expression increases the frequency of EB with endothelial sprouting and number of sprouts on each EB. These data suggest that E-cad, Cx43 and ZO-1 expression may act in a negative regulatory manner to inhibit both endothelial differentiation (more EB with sprouts) and proliferation (more sprouts per EB). Interestingly, disruption of ZO-1 patterning in human ESC has been reported to increase vasculogenesis in EB [33]. Expression of endothelial genes, Tie1 and Tie2, was not consistently altered in engineered ESC (data not shown). Tie1 and Tie2 levels in Kd-E-cad, Kd-Cx43 and Kd-ZO1 were similar to controls. Tie1 expression inhibits and Tie2 expression promotes angiogenesis [34]. The Tie1 results, which were not consistent with inhibition of angiogenesis, may be complicated by expression in lineages other than endothelial cells present in the EB [35]. Overall, however, the hematopoietic and endothelial data suggest that E-cad, Cx43 and ZO-1 expression influence lineage commitment at the point of endothelial and hematopoietic divergence (Figure 6A).

We demonstrate that ZO-2 levels are important for maintenance of early, intermediate and late stages of hematopoietic/endothelial differentiation in ESC. Kd-ZO2 ESC generated fewer Flk-1+ progenitors and hematopoietic cells than D3-ESC. Although endothelial sprouting in Kd-ZO2 ESC was similar to control ESC, there was an increase in number of sprouts on EB, suggesting increased proliferation of endothelial cells. These data suggest that low ZO-2 levels induce a block in ESC differentiation to Flk-1+ cells, but do not perturb endothelial commitment (Figure 6A). Although Flk-1 expression is necessary for hematopoietic and endothelial differentiation, Flk-1 expression has been observed in smooth muscle cells [36], cardiomyocytes [37], retinal progenitors [38] and other neural lineages [39]. Thus, a reduction in the frequency of Flk-1+ cells without alterations in endothelial commitment may suggest that endothelial cells can be generated through alternative non-Flk-1+ populations.

Overall, our data suggest that expression of junction molecules and their downstream components is associated with ESC commitment decisions between hematopoietic and endothelial lineages. Reduced expression of transmembrane junction molecules, E-cad and Cx43, increased endothelial commitment and decreased hematopoietic development, suggesting that these AM influence a common progenitor and activate divergent pathways towards lineage commitment (Figure 6B). Intracellular components, ZO-1 and ZO-2 possibly regulate differentiation decisions through additional interactions (Figure S4). For example, ZO-1 associates with components of adherens, gap and tight junction pathways, including direct interactions with α-catenin and connexins [16], [40] as well as claudins, ZO-2, JAM1, ZONAB, occludens and actin [14]. Expression of ZO-2 may be necessary for commitment to Flk-1+ progenitors; whereas, ZO-1 expression regulates later development decisions through ZONAB and α-catenin interactions. Modulation of cell fate commitment decisions between hematopoietic and endothelial lineages may occur through activation of intracellular signaling pathways, proliferation of endothelial progenitors or apoptosis of hematopoietic progenitors during EB development. Future analysis will require identification of downstream targets of these AM pathways in regulating hematopoietic/endothelial cell fate decisions and proliferation/apoptosis of hematopoietic/endothelial cells during EB development.

Finally, our data also suggest the AM may be useful as discriminants of transitional subpopulations within the Bry, Flk-1 and Scl expressing (Figure 6B) populations. Undifferentiated ESC express E-cad, Cx43, ZO-1 and ZO-2. As Bry transcription was up-regulated in mesoderm cells, E-cad and ZO-2 expression was down-regulated in the Bry+ cells, while Cx43 and ZO-1 levels were constant with the Bry- population of cells. Flk-1+ hematopoietic and endothelial progenitors had higher levels of ZO-1 and ZO-2, retained similar levels of Cx43 and reduced levels of E-cad than Flk-1- subpopulation. Scl+ cells, which have committed to the hematopoietic lineage, expressed ZO-1 in addition to the up-regulation of E-cad, Cx43 and ZO-2. These data not only suggest that E-cad, Cx43, ZO-1 and ZO-2 expression is associated with commitment decisions, but also that these markers identify subpopulations within the Bry+, Flk-1+ and Scl+ populations.

## Materials and Methods

### ESC Maintenance and EB Formation

Murine ESC lines D3-ESC [41], Bry-GFP [22] and Scl-LacZ [42] were cultured on mitomycin C treated STO cells (ATCC, Manassas, VA) as described [43]. ESC medium was exchanged daily during maintenance and pre-differentiation. EB were induced by culturing pre-differentiated ESC (2000–8000 cells/mL) in EB Medium as previously described [43] for 4 to 14 days at 37°C, 5% CO_2_. Briefly, EB medium consisting of 1% methylcellulose (Sigma, St. Louis, MO) in Iscove's modified Dulbecco's medium (IMDM; Mediatech, Herndon, VA) supplemented with 15% FBS, 2 mM L-glutamine (Mediatech, Herndon, VA), 450 µM MTG, 200 µg/mL transferrin (Sigma, St. Louis, MO) and 50 µM ascorbic acid (Sigma, St. Louis, MO) was used for undirected hematopoietic and endothelial differentiation analysis. EB cultures were supplemented with freshly prepared EB medium at days 4, 8 and 12 of differentiation induction.

### RNA Extraction and cDNA Synthesis

RNA was extracted from cells using Tri Reagent (Molecular Research Center, Cincinnati, OH) following manufacturer's recommendations. cDNA was synthesized using M-MLV reverse transcriptase (Fisher, Pittsburgh, PA) and random hexamers (Fisher, Pittsburgh, PA) for priming. Reverse transcription was completed by incubation at 25°C for 10 minutes followed by 37°C for 60 minutes. The enzyme was heat inactivated at 70°C for 10 minutes.

### Quantitative PCR

Quantitative PCR (qPCR) was performed using Brilliant SYBR Green qPCR Master Mix (Stratagene, La Jolla, CA) on a MX3000P qPCR System (Stratagene, La Jolla, CA) as follows: 95°C for 10 minutes; 45 cycles of 95°C for 30 seconds, 55°C for 30 seconds and 72°C for 30 seconds. The dissociation curve was analyzed at 95°C for 1 minute, 55°C for 30 seconds and ramping to 95°C. Relative quantification was determined with ΔΔCt analysis using GAPDH and untreated sample expression levels. Primers were designed to encompass multiple exons when possible to prevent interference from genomic DNA (Table S1).

### shRNA Expression Vector Construction and Transfection

Five siRNA sequences were designed for each target (E-cad, Cldn4, Cx43, ZO-1 and ZO-2) using siDesign Center (Dharmacon; http://www.dharmacon.com/sidesign/default.aspx) based on GenBank mRNA sequences. Sense and antisense siRNA sequences were converted to shRNA cassettes as reported [44]. shRNA cassettes were ligated to entry vector, pEN-mH1c (ATCC; Manassas, VA) at BamHI and XhoI sites. shRNA clones were transfected into D3-ESC (ATCC; Manassas, VA) with Lipofectamine 2000 (Invitrogen, Carlsbad, CA) according to manufacturer's recommendations.

### Lentiviral Preparation and Transduction

pEN-mH1c-shRNA constructs with silencing ability were subcloned into pDSL-hpUG (ATCC, Manassas, VA) destination vector using Gateway® LR Clonase™ (Invitrogen, Carlsbad, CA) according to manufacturer's recommendations. 293FT cells (Invitrogen, Carlsbad, CA) were transfected overnight using Lipofectamine 2000: 1 µg pDSL-hpUG-shRNA and 3 µg ViraPower Packaging Mix (Invitrogen, Carlsbad, CA). ESC were transduced with viral particles overnight at 37°C, 5% CO_2_ in ESC media supplemented with 5 µg/ml hexadimethrine bromide (Sigma, St. Louis, MO). Transduced cells were selected by sorting GFP+ cells on the FACSAria (Becton Dickinson, San Jose, CA). Target silencing was validated by RT-qPCR using SYBR Green reagents (Stratagene, La Jolla, CA).

### Colony Forming Cell Assay

EB were collected from methylcellulose media by dilution with IMDM, 2% FBS and centrifugation. Single cell suspensions were obtained by incubating cells at 37°C with 0.25% Trypsin/2.21 mM EDTA (Mediatech, Herndon, VA). EB cells were assayed for colony forming potential as described [8].

### Endothelial Sprouting Assay

Endothelial sprouting assay was performed as described [32]. Briefly, 1×10^4^ ESC were seeded in 35 mm dishes in 1.2 mg/ml collagen, type I (BD Bioscience, San Jose, CA) neutralized with 0.1 N NaOH. Collagen media contained 15% FBS, 450 µm MTG, 10 µg/ml insulin (Sigma, St. Louis, MO), 50 ng/ml human vascular endothelial growth factor (VEGF; Peprotech, Rocky Hill, NJ), 100 ng/ml human basic fibroblast growth factor (FGF-2; Peprotech, Rocky Hill, NJ) in IMDM. Cells were incubated at 37°C, 5% CO_2_. At day 6, 200 µl media without collagen was added and EB were analyzed on day 11. Image analysis was performed using AxioVision LE Software (Zeiss, Thornwood, NY).

### Flow Cytometry

EB were collected by centrifugation and single cells were obtained by trypsinization or treatment for 20 minutes at 37°C in 0.125% collagenase (Sigma, St. Louis, MO), 0.125% dispase (Worthington Biochemicals, Lakewood, NJ), and 1% DNase (Thermo Scientific, Waltham, MA) in IMDM, 10% FBS and manual dissociation. LacZ expression in Scl-LacZ cells was determined by flow cytometry with fluoroscein di-β-D-galactopyranoside (FDG; Invitrogen, Carlsbad, CA) according to manufacturer's recommendations. Briefly, cells were exposed to 300 µM chloroquine diphosphate (Sigma, St. Louis, MO) in ESC medium at 37°C for 20 minutes. Cells were incubated on ice with 1 mM FDG for 1 minute. FDG loading was terminated with 10 volumes ice cold ESC medium with 300 µM chloroquine diphosphate. Cells were exposed for 1 hour on ice in 2% BSA to the following antibodies: phycoerythrin (PE)-conjugated antibody to SSEA-1 (Chemicon, Temecula, CA), PE-conjugated antibody to Flk-1 (BD Bioscience, San Jose, CA), fluoroscein isothiocyanate (FITC)-conjugated antibody to E-cadherin (BD Bioscience, San Jose, CA) and PerCP-Cy5.5 conjugated antibody to CD45 (BD Bioscience, San Jose, CA). Dead cells were excluded using 2 µg/ml propidium iodide (Sigma, St. Louis, MO) or 0.5 µg/ml 4′,6-diamidino-2-phenylindole (DAPI; Sigma, St. Louis, MO). Cells were analyzed or sorted by FACSAria and LSRII (Becton Dickinson, San Jose, CA).

## Supporting Information

Figure S1 **shRNA sequences were cloned into Gateway® Entry and Destination vectors.** Lentiviral constructs constitutively expressing shRNA sequences were generated by cloning shRNA sequences into BamHI and XhoI sites of A) pEN-mH1c vector containing recombination sites (attL1 and attL2) flanking ubiquitously expressed mouse H1 promoter (mH1). Recombination between attL1/attL2 and attR1/attR2 on B) pDSL-hpUG lentiviral destination vector results in C) mH1 and shRNA incorporation into lentiviral plasmid with fluorescent reporter, GFP, constitutively expressed under Ubi-c promoter.
(TIF)

Figure S2 **Pluripotency Maintained in Knockdown ESC lines.** A) D3-ESC transduced with lentiviral constructs constitutively expressing GFP and shRNA specific to E-cadherin (Kd-E-Cad), Cx43 (Kd-Cx43), ZO1 (Kd-ZO1) and ZO2 (Kd-ZO2). GFP fluorescence was quantified using flow cytometry. Non-viable cells were excluded using DAPI. B) Cells were stained with SSEA-1 as a marker of pluripotency. Non-viable cells were excluded using DAPI. C) RNA extraction and quantitative PCR was performed on GFP expressing cells for Oct-4 expression and normalized to GAPDH. Expression levels reported relative to control D3-ESC. Error bars represent standard error of the mean for n = 4 samples.
(TIF)

Figure S3 **Protein levels of adhesion molecules are reduced in engineered ESC.** A) Western blot analysis was performed on knockdown ESC lines with antibodies specific to E-cad, Cx43, ZO-1 and Actin to determine B) ratio of protein expression to Actin compared with control D3-ESC (dashed line). C) RNA extraction and quantitative PCR was performed on Kd-ESC lines and Scram-GFP ESC to determine relative expression levels of E-cad, Cx43, ZO-1 and ZO-2. Samples were normalized to GAPDH and expressed relative to D3-ESC level (dashed line).
(TIF)

Figure S4 **Junction molecules interact across multiple pathways.** Cross-talk between gap, tight and adherens junction pathways occur through intracellular components, such as ZO-1. Association of ZO-1 with claudins (tight junctions) isolate transcription factors, such as ZONAB, from translocation into the nucleus where regulation of genes associated with cell growth and differentiation occurs. ZO-1 has binding domains for ZO-2, Connexins (gap junctions) and α-catenin (adherens junction). Interaction with α-catenin prevents assembly of cadherin/catenin complex, allowing intracellular accumulation of β-catenin, which translocates across the nuclear membrane to modulate expression of genes regulating cell growth and differentiation.
(TIF)

Table S1 **Adhesion molecule targets.** 21 genes associated with membrane or adhesion were chosen from reports of differential expression between pluripotent ESC and differentiated cells. Primers were designed between exons when possible for each gene. (CAM = Cell Adhesion Molecule; ECM = Extracellular Matrix; HL = Hematopoietic Lineage; JM = Junction Molecule; R-L = Receptor-Ligand; MA = Membrane Associated).
(DOC)
