# Supplementary figures and images for: Differential Adhesion Molecule Expression during Murine Embryonic Stem Cell Commitment to the Hematopoietic and Endothelial Lineages
Source: PLoS One. 2011 Sep 6;6(9):e23810. doi: 10.1371/journal.pone.0023810 (PMC3167810; doi:10.1371/journal.pone.0023810)

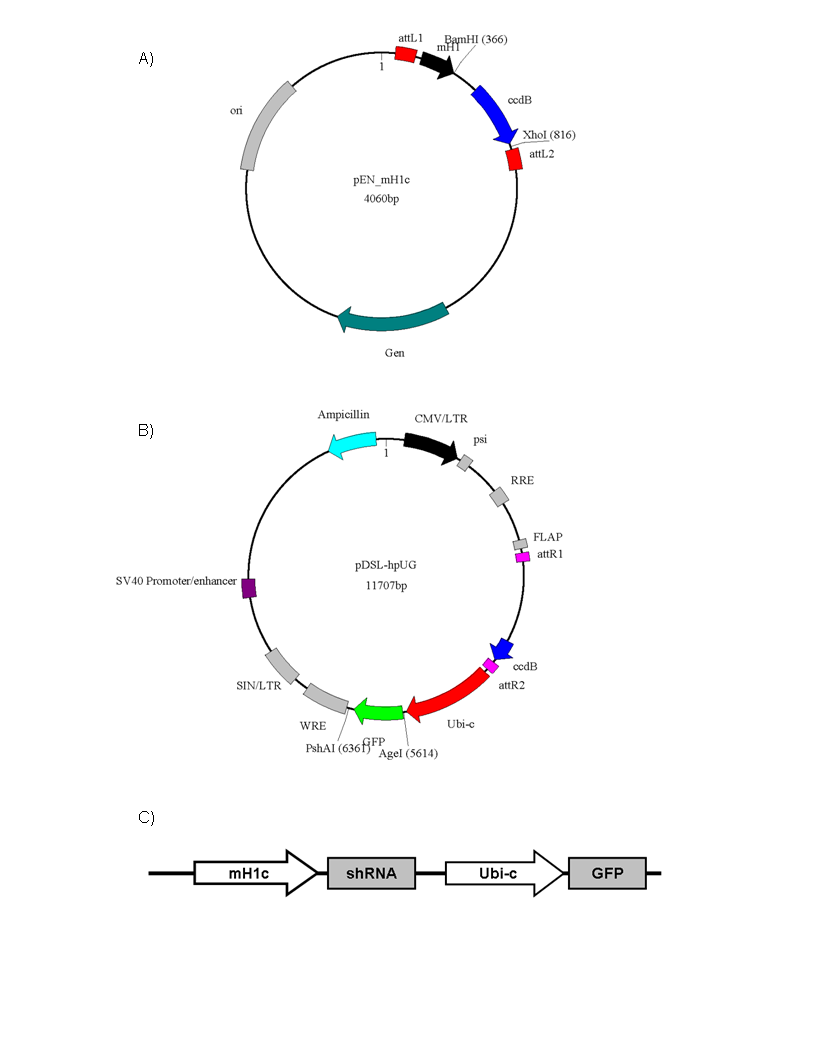

Supplement: Figure S1 — shRNA sequences were cloned into Gateway® Entry and Destination vectors. Lentiviral constructs constitutively expressing shRNA sequences were generated by cloning shRNA sequences into BamHI and XhoI sites of A) pEN-mH1c vector containing recombination sites (attL1 and attL2) flanking ubiquitously expressed mouse H1 promoter (mH1). Recombination between attL1/attL2 and attR1/attR2 on B) pDSL-hpUG lentiviral destination vector results in C) mH1 and shRNA incorporation into lentiviral plasmid with fluorescent reporter, GFP, constitutively expressed under Ubi-c promoter. (TIF) [file pone.0023810.s001.tif]

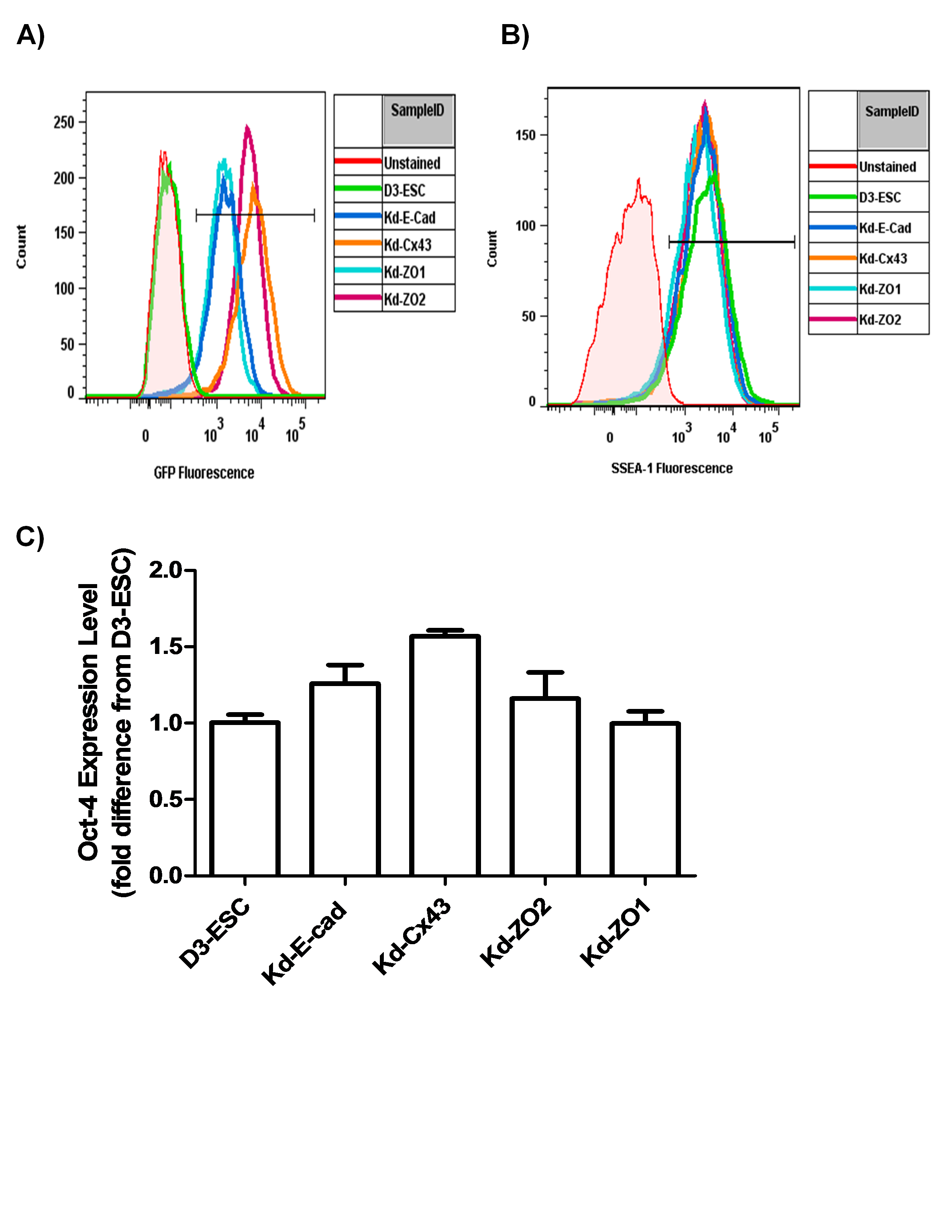

Supplement: Figure S2 — Pluripotency Maintained in Knockdown ESC lines. A) D3-ESC transduced with lentiviral constructs constitutively expressing GFP and shRNA specific to E-cadherin (Kd-E-Cad), Cx43 (Kd-Cx43), ZO1 (Kd-ZO1) and ZO2 (Kd-ZO2). GFP fluorescence was quantified using flow cytometry. Non-viable cells were excluded using DAPI. B) Cells were stained with SSEA-1 as a marker of pluripotency. Non-viable cells were excluded using DAPI. C) RNA extraction and quantitative PCR was performed on GFP expressing cells for Oct-4 expression and normalized to GAPDH. Expression levels reported relative to control D3-ESC. Error bars represent standard error of the mean for n = 4 samples. (TIF) [file pone.0023810.s002.tif]

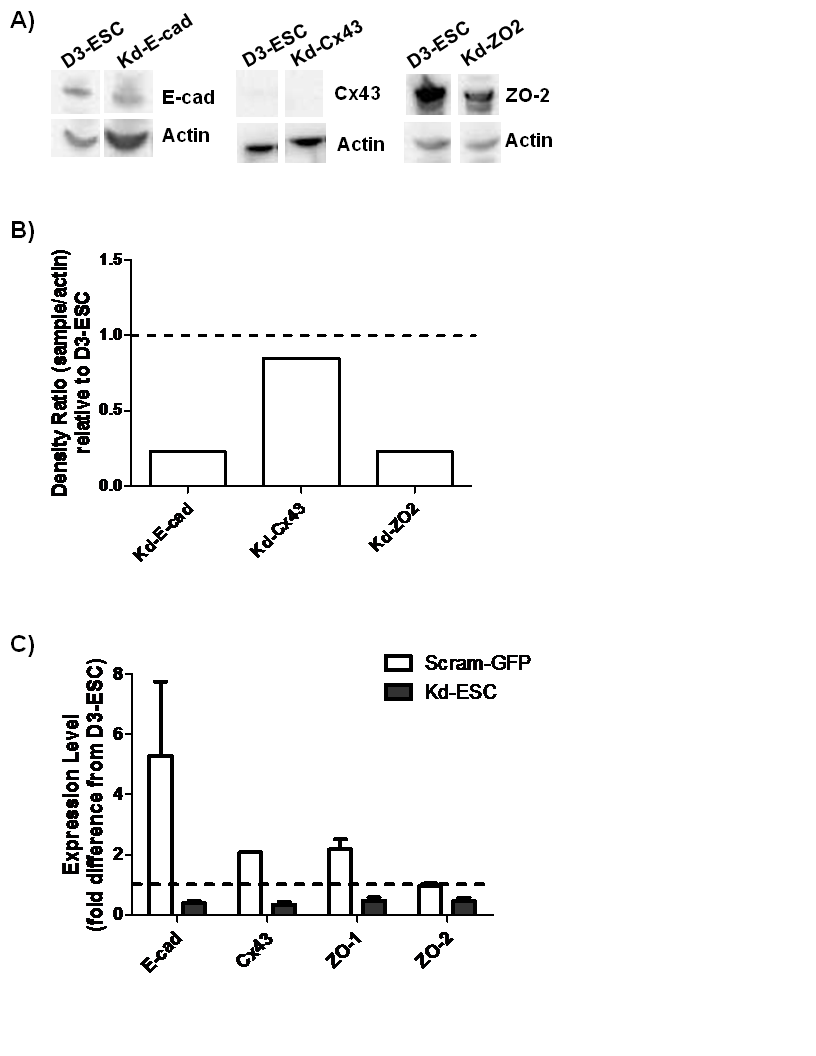

Supplement: Figure S3 — Protein levels of adhesion molecules are reduced in engineered ESC. A) Western blot analysis was performed on knockdown ESC lines with antibodies specific to E-cad, Cx43, ZO-1 and Actin to determine B) ratio of protein expression to Actin compared with control D3-ESC (dashed line). C) RNA extraction and quantitative PCR was performed on Kd-ESC lines and Scram-GFP ESC to determine relative expression levels of E-cad, Cx43, ZO-1 and ZO-2. Samples were normalized to GAPDH and expressed relative to D3-ESC level (dashed line). (TIF) [file pone.0023810.s003.tif]

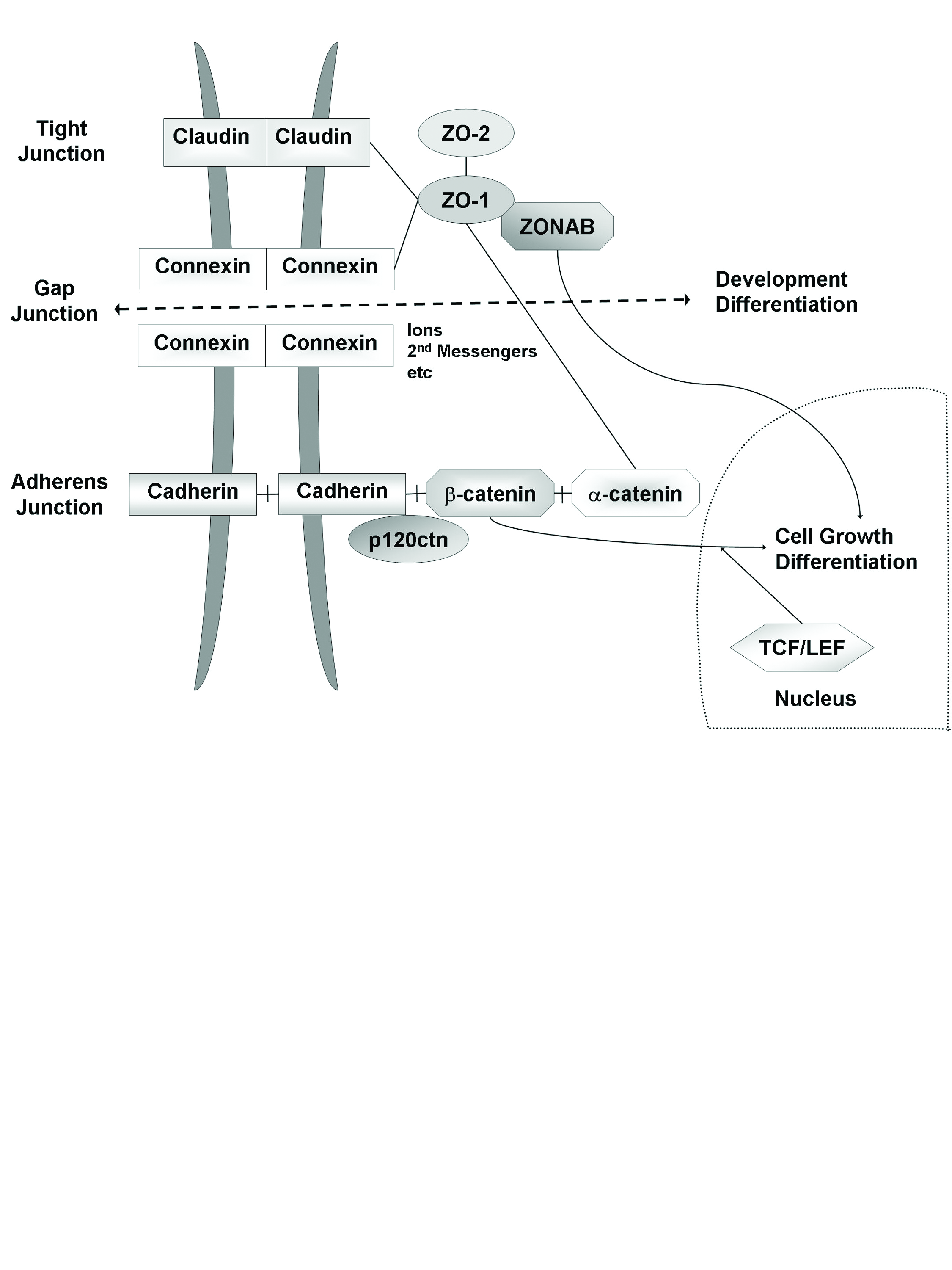

Supplement: Figure S4 — Junction molecules interact across multiple pathways. Cross-talk between gap, tight and adherens junction pathways occur through intracellular components, such as ZO-1. Association of ZO-1 with claudins (tight junctions) isolate transcription factors, such as ZONAB, from translocation into the nucleus where regulation of genes associated with cell growth and differentiation occurs. ZO-1 has binding domains for ZO-2, Connexins (gap junctions) and α-catenin (adherens junction). Interaction with α-catenin prevents assembly of cadherin/catenin complex, allowing intracellular accumulation of β-catenin, which translocates across the nuclear membrane to modulate expression of genes regulating cell growth and differentiation. (TIF) [file pone.0023810.s004.tif]
