# Supplementary material for: Differential Adhesion Molecule Expression during Murine Embryonic Stem Cell Commitment to the Hematopoietic and Endothelial Lineages
Source: PLoS One. 2011 Sep 6;6(9):e23810. doi: 10.1371/journal.pone.0023810 (PMC3167810; doi:10.1371/journal.pone.0023810)
Supplement: Table S1 — Adhesion molecule targets. 21 genes associated with membrane or adhesion were chosen from reports of differential expression between pluripotent ESC and differentiated cells. Primers were designed between exons when possible for each gene. (CAM = Cell Adhesion Molecule; ECM = Extracellular Matrix; HL = Hematopoietic Lineage; JM = Junction Molecule; R-L = Receptor-Ligand; MA = Membrane Associated). (DOC) [file pone.0023810.s005.doc]

| **Gene** | **Alternate Name** | **Function** | **Ref** | **5' Primer** | **3' Primer** |
| --- | --- | --- | --- | --- | --- |
| ICAM1 | Intercellular Cell Adhesion Molecule | CAM | [17] | TGGTGATGCTCAGGTATCCA | GGTGCCACAGTTCTCAAAGC |
| COL4A1 | Collagen, Type IV | ECM | [17] | TATGTCCAAGGCAACGAGC | AACCGCACACCTGCTAATG |
| NID1 | Nidogen | ECM | [17] | TTGGAGGCATCATCGGAT | ACAGTTCGGTGTAGGGCTCA |
| THBS1 | Thrombospondin I | ECM | [17] | CGATGGAGATGGAATCCTCA | TGGTTGGCATTAGGCACATAG |
| CD44 | CD44 | HL | [17] | GGAACTGGGAAACAAACCG | GTTGGCATCATCATCCATCA |
| CD81 | CD81 | HL | [20] | GCACATCCACATCAGCAGAT | CCACATTTCCTTCTATGAACCC |
| LY75 | Lymphocyte Antigen 75 | HL | [17] | AAGAAGCCACTGAACAACACG | AACCACCTCCACATCTGCTAA |
| CDH1 | E-cadherin | JM | [20] | CTGCCACCAGATGATGATACC | CGAACACCAACAGAGAGTCGTA |
| CLDN4 | Claudin-4 | JM | [17] | GGAATCTCCTTGGCAGTCCT | CACCCACGATGATGCTGAT |
| CLDN6 | Claudin-6 | JM | [17] | TGTGTGGTTCAGAGCACTGG | AGCAGACAGGAATGAGCGTC |
| GJA1 | Connexin-43 | JM | [16] | GGATTGAAGAACACGGCAA | AGAGCGAGAGACACCAAGGA |
| GJB3 | Connexin-31 | JM | [17] | ATTGAGTGGCGTGAACCAG | TGACCAGCATAGACGGACAC |
| TJP1 | Zona Occludens-1 | JM | [20] | AGCGATTCAGCAGCAACA | GGCTCAGAGGACCGTGTAAT |
| TJP2 | Zona Occludens-2 | JM | [20] | GGAAGATGTGCTCCATTCG | TGCCGACTCCTCTCACTGTA |
| DDR1 | Discoidin Domain Receptor Kinase | R-L | [17] | CACCATTCCTGACAGCGAT | GGAGTAACGCAACCGATAGC |
| ITGB4 | Integrin, beta-4 | R-L | [20] | AAGCAGAAGAGGTGGTGGAGTA | AGCAGAGCAGCAGAAGCAA |
| KIT | c-Kit Receptor | R-L | [17] | CAGGAGCAGAGCAAAGGTGTA | CGACCACAAAGCCAATGAG |
| WNT3 | Wingless-type MMTV Integration Site | R-L | [17] | GCACAACAATGAAGCAGGC | ATCTCGGAGGCACTGTCGTA |
| ABCG2 | ATP-binding cassette, sub-family G | MA | [16] | TATGATGCCGCTGGAATG | CCACCGTCTTCTTCAGTCCT |
| APP | Amyloid Precursor Protein | MA | [17] | CACCACAACCACCACTGAGT | AAGTTCTTGGCTTGACGCTC |
| BIN1 | Amphiphysin 2 | MA | [17] | GAGCAACACCTTCACAGTCAAG | CAGGGACAAATGCGTCATC |
